# PP78 Procedures And Results Of A Drug Assessment Sector In A Hospital-Based Health Technology Assessment Unit At A Tertiary Pediatric Hospital

**DOI:** 10.1017/S0266462325102961

**Published:** 2025-12-29

**Authors:** Marcela Rousseau, Graciela Demirdjian

## Abstract

**Introduction:**

The Hospital-Based Health Technology Assessment (HB-HTA) Unit at the third-level pediatric Hospital Garrahan has had a specific Drug Assessment Sector since 2019 for mandatory evaluation of all incorporation requests for high-cost or high-risk medications. The aim of this study was to analyze its production and response times, the annual cost per patient of drugs assessed, the proportion of recommendations to reject, management acceptance, and patient results of drugs incorporated during the last five years.

**Methods:**

All medication incorporation requests follow a standardized procedure. The Pharmacy and Therapeutics Committee makes an initial appraisal of relevance. Drugs with a high cost, safety risk, or any significant organizational impact are submitted to the HB-HTA Unit for systematic review. A non-binding report is sent to the Executive Director to support decision-making. The Drug Sector also carries out proactive assessments like yearly analyses of pharmacy medication expenses, drug use variability, overutilization, trends in consumption, interchangeability, and pharmacovigilance. The proportion of full and rapid reports, rejection rate, response time, and annual cost per patient were analyzed.

**Results:**

In five years, the Drug Assessment Sector at the HB-HTA Unit produced 24 reports (56% were full reports and 44% were brief or rapid). Of these, 76 percent were for high-cost drugs and 96 percent were accepted by management. The median response time was 106 days (range 5 to 511). The recommendations included approvals for restricted conditions (36%) and rejections for safety issues, lack of evidence of effectiveness, or high budget impact (21%). The annual estimated cost per patient of assessed drugs ranged from ARS10,528 to ARS304,837,050 (USD1,757 to USD314,265). Follow-up for a minimum of two years included six drugs (44 patients), which showed compliance with restricted use recommendations and no clinical results justifying disinvestment.

**Conclusions:**

The creation of a specific Drug Assessment Sector in the HB-HTA Unit at our hospital has produced a more rational incorporation and utilization of high-cost drugs. Variable response times result from urgency and coexisting requests. Despite the non-binding recommendations, management acceptance was high. Follow-up and clinician reports of patient results enable review of the decisions made.

